# *NEXMIF* pathogenic variants in individuals of Korean, Vietnamese, and Mexican descent

**DOI:** 10.1002/ajmg.a.62686

**Published:** 2022-02-10

**Authors:** Elizabeth Langley, Laura S. Farach, Mary K. Koenig, Hope Northrup, David F. Rodriguez‐Buritica, Kate Mowrey

**Affiliations:** ^1^ Division of Medical Genetics, Department of Pediatrics McGovern Medical School at the University of Texas Health Science Center at Houston (UTHealth Houston) and Children's Memorial Hermann Hospital Houston Texas USA; ^2^ Division of Child and Adolescent Neurology, Department of Pediatrics McGovern Medical School at the University of Texas Health Science Center at Houston (UTHealth Houston) and Children's Memorial Hermann Hospital Houston Texas USA

**Keywords:** autism, epilepsy, *KIAA2022*, *NEXMIF*, X‐linked intellectual disability

## Abstract

*NEXMIF* pathogenic variants have been known to produce a wide spectrum of X‐linked intellectual disability (ID) in both males and females. Thus far, few individuals from diverse populations have been described with NEXMIF‐related disorders. Herein, we report three individuals with *NEXMIF* pathogenic variants, the first two are the only males of Korean and Vietnamese descent described with this disorder to our knowledge. The last patient is a Hispanic female who harbors the same pathogenic variant as a previously described Caucasian individual, but with differing clinical presentation. These patients present with many classic symptoms of NEXMIF‐related disorders including ID, epilepsy, developmental delay, and dysmorphic features. In addition, they have symptoms that have not been thoroughly described in the literature, including allergies with multiple anaphylactic events and hypothyroidism. This report is intended to raise awareness and educate about the clinical signs that may prompt testing for NEXMIF‐related disorders.

## 1 INTRODUCTION

X‐linked intellectual disability (XLID) accounts for approximately 10% of intellectual disability (ID) in males and involves over 100 genes (Van Maldergem et al., 2013). *NEXMIF*, also known as *KIAA2022*, is a gene located at Xq13.2 that is widely expressed in the brain and involved in neurite outgrowth and extension (Cantagrel et al., 2004). Its specific function is not fully known, however, pathogenic variants in *NEXMIF* result in XLID in males and some females in conjunction with other neurological and nonneurological problems. It was previously hypothesized that females were asymptomatic, but recent studies have shown symptomatic females harboring *NEXMIF* pathogenic variants have a wide spectrum of clinical presentations (Farach & Northrup, 2015). Typical features in males and females associated with NEXMIF‐related disorders include ID, epilepsy, delayed or absent speech, dysmorphic facial features, and delayed development (Van Maldergem et al., 2013). These disorders have been documented in few diverse populations, often without images. Further depictions of the disorder in individuals from diverse backgrounds may prove beneficial for identifying and diagnosing *NEXMIF* pathogenic variants in future patients. Herein, we present a biracial Korean male, Vietnamese male, and Hispanic female, the first two having novel *NEXMIF* pathogenic variants not previously reported, but with all three demonstrating characteristic phenotypes along with unique or underreported associated symptoms.

## 2 CASE REPORT

### 2.1 Patient 1

Patient 1 is a 13‐year‐old male, the second child of a nonconsanguineous Korean female and Caucasian male, who was born at an estimated 34 weeks via C‐section. There is limited information about prenatal history and family history secondary to his adoption on the second day of life. The patient's birth weight was reported to be 4620 g (>97th percentile). In infancy, he began developing symptoms of constipation, cyclic vomiting, rash, episodes of rigidity, hypotonia, and global motor delay. In his first years of life, he experienced strabismus that did not correct with patching, gastroparesis, eczema, ongoing vomiting and constipation, proteinuria, and numerous allergic and anaphylactic reactions. He was diagnosed with hypothyroidism at 2 years old. He was prescribed levothyroxine, and his hypothyroidism has since been well‐controlled. He demonstrated gross motor delay: rolled from stomach to back at 6 months, sat unassisted at 13 months, crawled at 21 months, and walked independently at 5 years. During these first few years, he underwent numerous tests and different treatments were tried to assist with vomiting, constipation, and allergies including coenzyme Q10, amino acid‐based formula, and bethanechol. Normal imaging included two upper endoscopies and colonoscopy at age 2 years, renal ultrasound at age 4 years, echocardiogram at ages 2 and 4 years, and brain MRI at ages 2 and 4 years.

**FIGURE 1:**
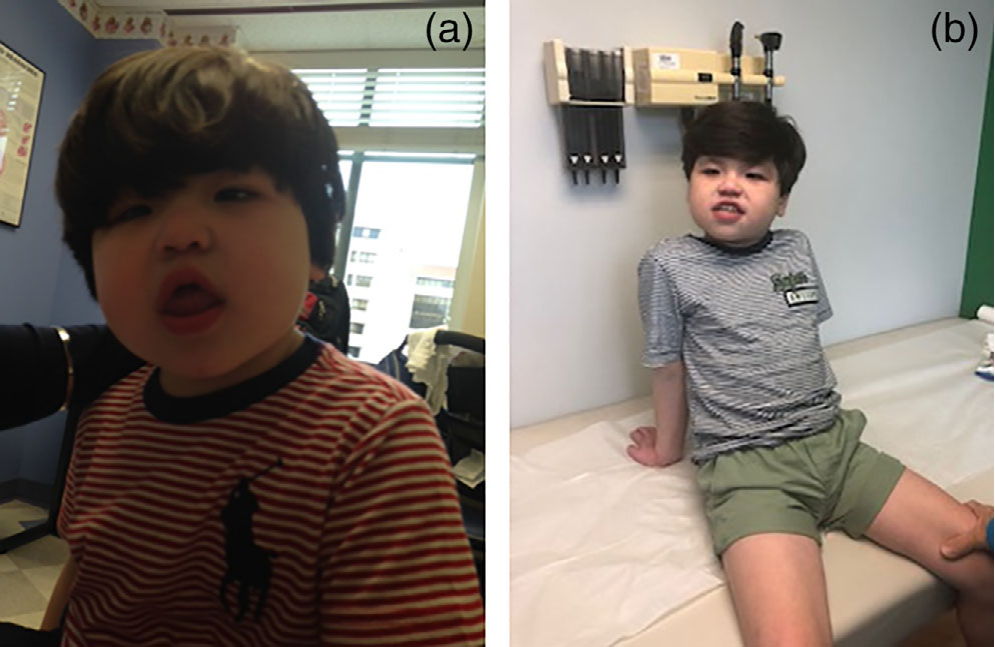
(a) Patient 1 at 6 years of age; strabismus and dysmorphic facial features including round face, short nose, and short philtrum can be appreciated. (b) Patient 1 at 12 years of age, dysmorphic features and short stature present

He is now age 13 years and nonverbal. He has been diagnosed with ID and autism and has recently developed tonic–clonic seizures which are not yet well‐controlled currently using levetiracetam. His last physical examination was at 12 years. His weight was 26 kg (<5th percentile, 50th for an 8‐year‐old) and height was 124 cm (<5th percentile, 50th for a 7‐and‐a‐half‐year‐old). His dysmorphic features include a round face, short nose, short philtrum, and esotropia (Figure 1a,b).

Genetic testing was first performed at 18 months, secondary to his motor delay and included a chromosome microarray (CMA), Fragile X, porphyria testing, *MECP2* sequencing and deletion/duplication analysis, Prader‐Willi DNA methylation, mitochondrial DNA testing, and a muscle biopsy—all of which were normal. At the age of 7 years, proband exome sequencing was performed and revealed that he has a hemizygous pathogenic variant (PSV1, PM2, PP4) c.788delC (p.T2631fsX41) in *NEXMIF* [NM_001008537.1] and a variant of uncertain significance (VUS) c.65T>C (p.V22A) in *SYP* [NM_003179.2] (Richards et al., 2015).

### 2.2 Patient 2

Patient 2 was the second child born to nonconsanguineous Vietnamese parents at term via repeat cesarean section. The pregnancy was complicated by intrauterine growth restriction. At birth, his weight was reported to be 2890 g (13th percentile). Birth length and head circumference were not reported. Otherwise, there were no neonatal concerns, and he was discharged at day of life 2.

At 6 months, he was evaluated by neurology for developmental delay. At that appointment, he was noted to be delayed in speech and motor skills as well as diffusely hypotonic, and obese. Notably, his hypotonia was more prominent axially and distally. A routine electroencephalogram (EEG) was performed at 7 months and was normal. Shortly thereafter, he started physical and occupational therapy, but was discontinued after approximately 40 visits secondary to the COVID‐19 pandemic. Speech therapy was never obtained by the family. Even after receiving physical and occupational therapy, he remained globally developmentally delayed with minimal improvement. He achieved head control at 15 months, rolled over stomach to back at 18 months, but was unable to roll over back to stomach at 20 months of age. At 20 months, he could coo, but was unable to babble. In addition, he exhibited early signs of autism including fixated interest, oral fixation, repetitive play, inability to soothe himself, and head banging, but had not been formally evaluated. He also had strabismus of the left eye, noted at 1 year of age by an ophthalmologist, that did not respond to patching and will need surgical intervention.

**FIGURE 2:**
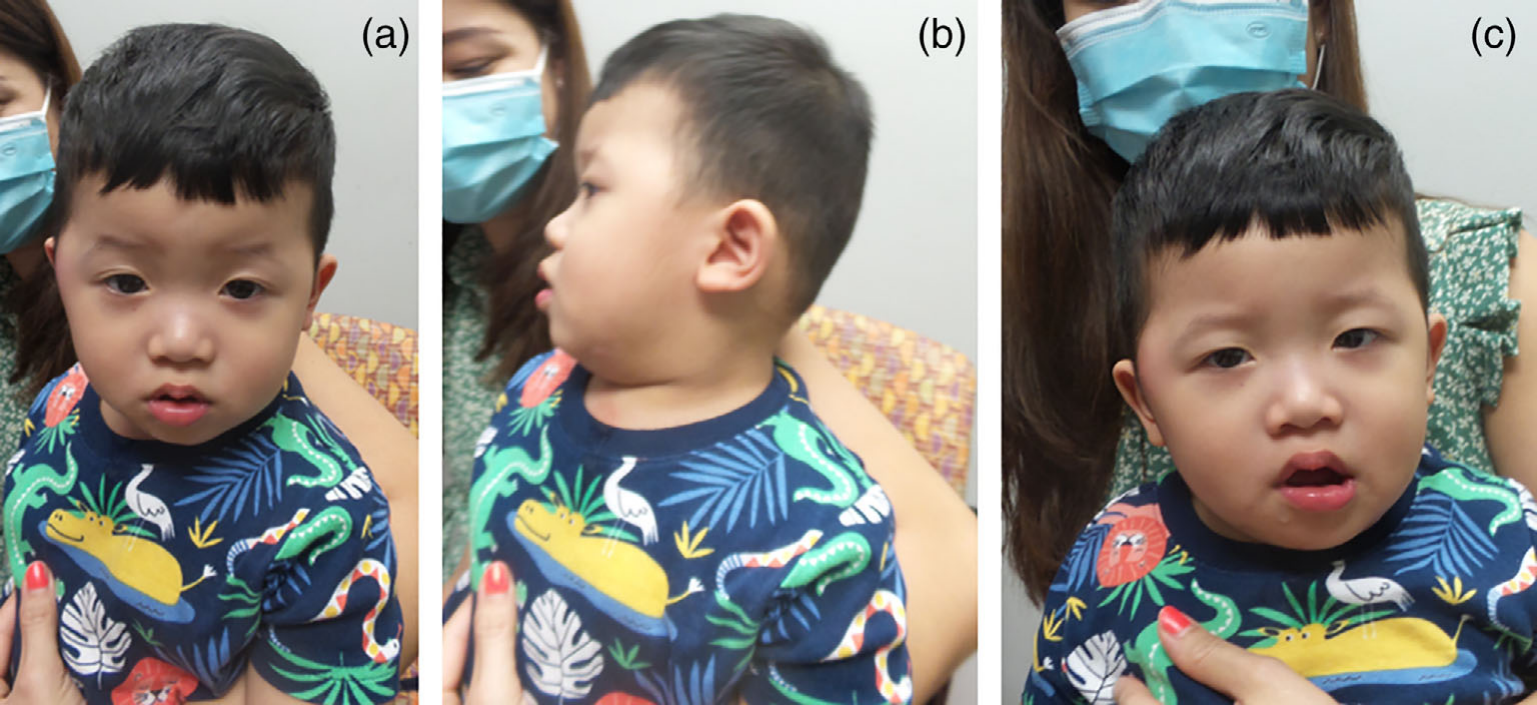
(a–c) Patient 2 at 20 months; strabismus and obesity observed

At 20 months, he had a genetics evaluation where he was noted to be nondysmorphic but was large for his age and had areas of hyperpigmentation on his abdomen, back, buttocks, knees, and torso, as well as a hyperpigmented macule behind his right ear (Figure 2a–c). His obesity persisted at 20 months with a weight of 14.81 kg (>97th percentile, 50th for a 3‐year‐old), height of 90 cm (>97th percentile, 50th for a 2‐and‐a‐half‐year‐old), and head circumference of 48 cm (50th percentile). CMA, Fragile X, and Prader‐Willi DNA methylation were normal. Trio, whole exome sequencing + mitochondrial sequencing revealed a hemizygous, de novo pathogenic variant (PVS1, PS2, PM2, PM6, PP4) c.846_849delTGTC (p.V283TfsX20) in *NEXMIF* [NM_001008537.1] (Richards et al., 2015). There was no family history of ID, seizures, or other medical problems associated with NEXMIF‐related disorders.

### 2.3 Patient 3

Patient 3 is a 6‐year‐old Hispanic female born vaginally to nonconsanguineous parents from Mexico at term after an uncomplicated pregnancy. At birth, she weighed 2920 g (17th percentile) and was noted to be mildly hypotonic. Otherwise, there were no neonatal concerns, and she was discharged at day of life 2.

Motor development was appropriate, but she did have a slight speech delay. At 16 months of age, she started having absence seizures characterized by eye rolling associated with loss of consciousness and head drops. Over the next few years, she failed therapy with multiple antiepileptic drugs (AEDs) including levetiracetam, clonazepam, topiramate, and ethosuximide. Her AED regimen at last evaluation was ethosuximide, topiramate, and cannabidiol oil and she was still experiencing 3–5 absence seizures per day. At the age of 6 years, a 43‐h long video EEG was abnormal due to the presence of intermittent brief, bilateral high amplitude bifrontal spike and polyspike wave discharges. A previous brain MRI at 2 years was normal. In addition to refractory epilepsy, her speech delay persisted, and at 6 years, she is unable to read, write, identify the alphabet, or colors, but can speak in full sentences and follow simple commands. She has dysmorphic features including a square face, long palpebral fissures, a short, smooth philtrum, and thin upper lip. In addition, she experiences behavioral disturbances including agitation, aggression, anxiety, tantrums, and attention deficit/hyperactive disorder. She also presents with bone pain and mild vitamin D deficiency. Her last vitamin D, 25‐OH level was 27 ng/ml without supplementation. Her mother also complains of the patient having a diminished appetite at times, possibly a side effect of topiramate, ethosuximide, or levetiracetam. At 6 years old, her weight is 19 kg (14th percentile), height is 111.2 cm (5th percentile), and head circumference is 51.4 cm (50–70th percentile). Given her history of seizures, she underwent an epilepsy panel at a commercial laboratory. A possibly mosaic, de novo pathogenic variant (PVS1 + PS2) c.937C>T (p.Arg333*) in *NEXMIF* (NM_001008537.2) was identified (Nykamp et al., 2017). This pathogenic variant was interpreted by the lab as being possibly mosaic due to an allele frequency of the variant between 7% and 17%. Additional tissues, such as hair or urine, were not collected to assess the level of mosaicism further in this patient. CMA and Fragile X were normal.

## 3 DISCUSSION

Here, we present three unrelated cases of NEXMIF‐related disorders in diverse populations. Unless ethnicity was unstated in previous case reports, to our knowledge, no other cases in Korean or Vietnamese individuals have been reported in the medical literature to date. In addition, the pathogenic variants described in Patients 1 and 2 are novel and have not been previously reported in other publications or publicly accessible databases.

For Patient 1, the phenotype of developmental delay, ID, strabismus, ASD, dysmorphic features, poor growth, and gastrointestinal problems aligns with the phenotype present in previously described males with NEXMIF‐related disorders (Van Maldergem et al., 2013). Furthermore, this patient's onset of epilepsy in late childhood is consistent with other cases in males. Females, such as Patient 3, tend to have an earlier onset of epilepsy and a higher frequency of epilepsy in comparison to males (Stamberger et al., 2021). Upon review of the literature, Patient 1 is the first to have such profound allergies with multiple anaphylactic events. Involvement of the endocrine and immune systems, as seen in this patient's hypothyroidism and allergies, may be unrelated but may also represent a new, yet unrecognized, component of NEXMIF‐related disorders. These findings indicate that *NEXMIF* protein may play a role in multiple areas of the body producing more multisystemic effects than previously recognized. It is important to acknowledge the presence of the VUS identified in *SYP* on whole exome sequencing. *SYP* is located at Xq11.23 and encodes for a protein that regulates synaptic vesicle endocytosis (Kwon & Chapman, 2011). This gene was clinically implicated when Tarpey et al. identified four families with XLID with variants in *SYP* that segregated with phenotype. Phenotype primarily includes males with ID and variable presence of epilepsy (Tarpey et al., 2009). The *SYP* variant in Patient 1 differs from those described and a recent verbal update by the commercial laboratory indicated this variant remains a VUS, thus the contribution of the variant to the patient's clinical picture is unknown. The possible association of severe allergies, anaphylaxis, and autoimmune problems and *NEXMIF* pathogenic variants warrants further investigation.

Patient 2, the first reported Vietnamese patient, displays many classic symptoms of NEXMIF‐related disorders including ID, strabismus, ASD, and developmental delays. In contrast to the poor growth reported with NEXMIF‐related disorders, this individual is obese. Although obesity is reported in adults with NEXMIF‐related disorders, it is not often observed in infants (Stamberger et al., 2021). Therefore, obesity in childhood may be a clinical feature underrepresented in this disorder. The finding may, however, be due to environmental factors such as overfeeding, but demonstrates that children with NEXMIF‐related disorders can present with overgrowth.

Few patients have been previously described in the literature that harbor identical pathogenic variants in *NEXMIF*. Not only does Patient 3 provide another example of a NEXMIF‐related disorder in a Hispanic female, but she also harbors the same pathogenic variant described in a Caucasian female, Patient 5, in Webster et al. (2017). Shared clinical features between our patient and Webster et al. (2017)'s Patient 5 include: anxiety, tantrums, refractory epilepsy, and absence of developmental motor delay (Webster et al., 2017). However, our patient has dysmorphic features including a square face, long palpebral fissures, a short, smooth philtrum, and a thin upper lip, while the previously reported patient is nondysmorphic. In addition, Webster's patient experienced gastroesophageal reflux in infancy, constipation, and encopresis (Webster et al., 2017), while our patient has not experienced any of these symptoms. These differing clinical presentations are evidence of the known variable expressivity in NEXMIF‐related disorders. Another layer of complexity regarding the phenotype of Patient 3 is due to the reported mosaicism.

Altogether, these patients describe how NEXMIF‐related disorders present in individuals from diverse backgrounds. Photographic documentation displaying differences in dysmorphic features, stature, and overall clinical presentation are used to expand the phenotype and raise awareness of clinical clues that may help lead to the diagnosis of a NEXMIF‐related disorder.

## CONFLICT OF INTEREST

The authors do not have any relevant conflicts of interest to disclose.

## AUTHOR CONTRIBUTIONS

**Elizabeth Langley:** Developed concept, performed chart review, and manuscript writing. **Laura Farach:** Developed concept, provided clinical information, manuscript writing. **Mary Kay Koenig:** Provided clinical information and manuscript editing. **Hope Northrup:** Provided clinical information and manuscript editing. **David Rodriguez‐Buritica:** Provided clinical information and manuscript editing. **Kate Mowrey:** Developed concept, performed chart review, manuscript writing.

## Data Availability

The data that support the findings of this study are available on request from the corresponding author. The data are not publicly available due to privacy or ethical restrictions.
